# Assessment of the bi-directional relationship between blood mitochondrial DNA copy number and type 2 diabetes mellitus: a multivariable-adjusted regression and Mendelian randomisation study

**DOI:** 10.1007/s00125-022-05759-6

**Published:** 2022-07-22

**Authors:** Wenyi Wang, Jiao Luo, Ko Willems van Dijk, Sara Hägg, Felix Grassmann, Leen M. `t Hart, Diana van Heemst, Raymond Noordam

**Affiliations:** 1 Department of Human Genetics, Leiden University Medical Center, Leiden, the Netherlands; 2 Department of Internal Medicine, Section of Gerontology and Geriatrics, Leiden University Medical Center, Leiden, the Netherlands; 3 Department of Clinical Epidemiology, Leiden University Medical Center, Leiden, the Netherlands; 4 Department of Internal Medicine, Division Endocrinology, Leiden University Medical Center, Leiden, the Netherlands; 5 Leiden Laboratory for Experimental Vascular Medicine, Leiden University Medical Center, Leiden, the Netherlands; 6 Department of Medical Epidemiology and Biostatistics, Karolinska Institute, Stockholm, Sweden; 7 Health and Medical University, Potsdam, Germany; 8 Department of Cell and Chemical Biology, Leiden University Medical Center, Leiden, the Netherlands; 9 Department of Biomedical Data Sciences, Molecular Epidemiology, Leiden University Medical Center, Leiden, the Netherlands; 10 Department of Epidemiology and Data Sciences, Amsterdam University Medical Center, Location VUmc, Amsterdam, the Netherlands

**Keywords:** Mendelian randomisation, Mitochondrial DNA copy number, Prospective analyses, Type 2 diabetes mellitus

## Abstract

**Aims/hypothesis:**

Mitochondrial dysfunction, which can be approximated by blood mitochondrial DNA copy number (mtDNA-CN), has been implicated in the pathogenesis of type 2 diabetes mellitus. Thus far, however, insights from prospective cohort studies and Mendelian randomisation (MR) analyses on this relationship are limited. We assessed the association between blood mtDNA-CN and incident type 2 diabetes using multivariable-adjusted regression analyses, and the associations between blood mtDNA-CN and type 2 diabetes and BMI using bi-directional MR.

**Methods:**

Multivariable-adjusted Cox proportional hazard models were used to estimate the association between blood mtDNA-CN and incident type 2 diabetes in 285,967 unrelated European individuals from UK Biobank free of type 2 diabetes at baseline. Additionally, a cross-sectional analysis was performed to investigate the association between blood mtDNA-CN and BMI. We also assessed the potentially causal relationship between blood mtDNA-CN and type 2 diabetes (*N*=898,130 from DIAGRAM, *N*=215,654 from FinnGen) and BMI (*N*=681,275 from GIANT) using bi-directional two-sample MR.

**Results:**

During a median follow-up of 11.87 years, 15,111 participants developed type 2 diabetes. Participants with a higher level of blood mtDNA-CN are at lower risk of developing type 2 diabetes (HR 0.90 [95% CI 0.89, 0.92]). After additional adjustment for BMI and other confounders, these results attenuated moderately and remained present. The multivariable-adjusted cross-sectional analyses showed that higher blood mtDNA-CN was associated with lower BMI (−0.12 [95% CI −0.14, −0.10]) kg/m^2^. In the bi-directional MR analyses, we found no evidence for causal associations between blood mtDNA-CN and type 2 diabetes, and blood mtDNA-CN and BMI in either direction.

**Conclusions/interpretation:**

The results from the present study indicate that the observed association between low blood mtDNA-CN and higher risk of type 2 diabetes is likely not causal.

**Graphical abstract:**

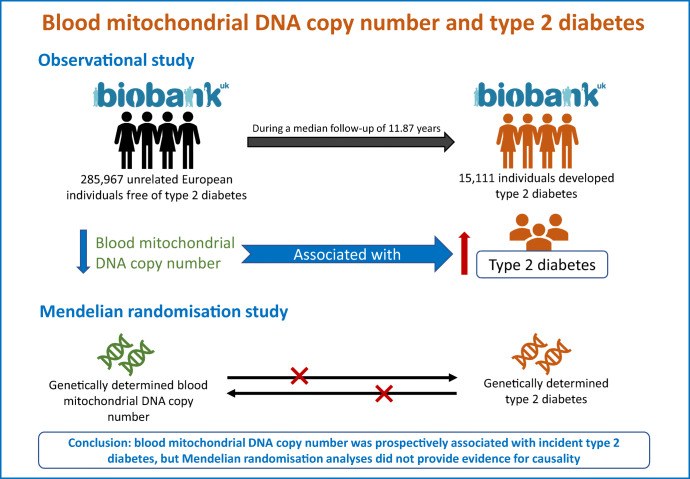

**Supplementary Information:**

The online version contains peer-reviewed and unedited supplementary material available at 10.1007/s00125-022-05759-6.

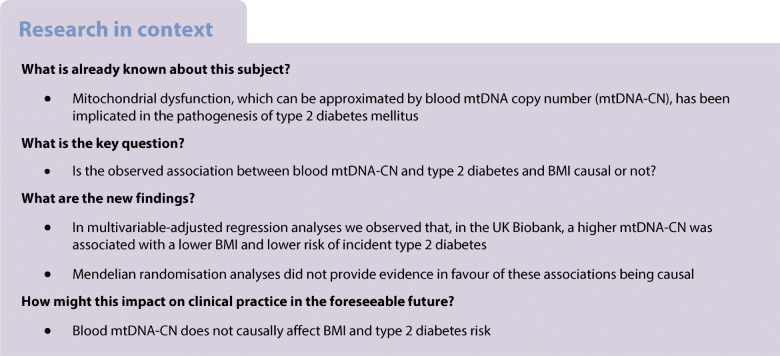



## Introduction

Type 2 diabetes mellitus, which is characterised by a combination of insulin resistance and insufficient insulin secretion, is a major contributor to the burden of morbidity and mortality worldwide [1]. Multiple risk factors have been associated with an increased risk of developing type 2 diabetes, including obesity, adverse lifestyle and genetic factors [2].

Mitochondria generate most of the cell’s need for chemical energy in the form of ATP, and mitochondrial dysfunction has been implicated in various aspects of the development and complications of type 2 diabetes including insulin resistance, obesity and beta cell dysfunction [3]. Mitochondrial DNA (mtDNA) is a circular and double-stranded DNA molecule comprising 37 genes, of which 13 genes are involved in the electron transport chains and generation of ATP to provide energy for cells, while the remaining genes encode proteins involved in the assembly of amino acids into functional proteins [4]. Although mitochondria have been implicated in the onset of type 2 diabetes, only a few of the rare mtDNA genetic variants are associated with diabetes mellitus [5].

MtDNA copy number (mtDNA-CN) is considered as a proxy for mitochondrial function [6]. Low peripheral blood mtDNA-CN has been associated with various age-related diseases, including type 2 diabetes, CVD and cancer [7–9]. A lower mtDNA-CN has also been observed in skeletal muscle and adipose tissue of individuals with obesity or type 2 diabetes [10, 11]. Similarly, a lower mtDNA-CN in beta cells has also been associated with decreased beta cell function [12].

Thus far, epidemiological studies investigating the relationship between mtDNA-CN and type 2 diabetes are limited. To the best of our knowledge, only four cohort studies have investigated the association between blood mtDNA-CN and type 2 diabetes, among which three were conducted using a prospective study design [13–16]. Two out of the four studies showed that low blood mtDNA-CN was associated with an increased risk of developing type 2 diabetes. However, these studies were performed in a relatively small population with a low number of (incident) type 2 diabetes cases, which warrants replication of the findings in larger study samples. In addition, none of these studies have examined the question whether the relationship between blood mtDNA and type 2 diabetes is causal. Therefore, the aim of our study was to investigate the association between blood mtDNA-CN and incident type 2 diabetes using data from the UK Biobank (UKB). Additionally, the bi-directional relationship between blood mtDNA-CN and type 2 diabetes and BMI was assessed by bi-directional two-sample Mendelian randomisation (MR) analyses [17].

## Methods

### Prospective analyses

#### Study population

The data used in the present study were derived from the UKB. The UKB is a large-scale prospective cohort study containing in-depth genetic and health information from over 500,000 participants aged 40–70 years at recruitment. Participants were recruited between 2006 and 2010 at 22 assessment centres across the UK. Baseline examinations in all participants included physical measurements (e.g., BP), lifestyle and environmental information (e.g., diet, exercise, smoking, alcohol), biological sampling (e.g., blood and urine), medical history, etc. The UKB study was approved by the North West Multi-centre Research Ethics Committee. Access to information to invite participants was approved by the Patient Information Advisory Group for England and Wales, and the National Information Governance Board for Health and Social Care. All participants in the UKB study provided written informed consent, and detailed research ethics approval can be found on the official website (https://www.ukbiobank.ac.uk/learn-more-about-uk-biobank/about-us/ethics). The present study was conducted using accepted proposal 22474.

The genotype data released from UKB contain genotypes of 488,377 participants from diverse ancestries. A total of 195,203 participants were removed based on the following exclusion criteria: (1) samples did not meet the quality control criteria (*N*=81,552); (2) participants were related (*N*=38,642); (3) participants were from non-European ancestry (*N*=65,498); (4) informed consent was withdrawn (*N*=71); and (5) SD of autosomal probes (last step of mtDNA-CN calculation) was greater than 0.37 (*N*=9,440). More details on exclusion criteria can be found elsewhere [18]. After filtering, a total of 293,174 unrelated European participants were included in the present study.

#### Computation of mtDNA copy number in blood

Blood mtDNA-CN, the exposure of interest, was calculated based on the intensities of genotyping probes on the mitochondrial chromosome on the Affymetrix array in the UKB participants. The pipeline for calculating mtDNA-CN from intensities of probes mapping to the mitochondrial genome has been described in detail previously [18, 19]. Briefly, the relative amount of mtDNA hybridised to the array at each probe was the log_2_ transformed ratio (L2R) of the observed genotyping probe intensity divided by the reference intensity. Initial values of mtDNA-CN were computed as the median L2R values across all 265 variants passing quality control on the mitochondrial DNA. The single mtDNA-CN estimated for each individual was derived by computation of weighted mtDNA-CN by using initial values and normalisation of mtDNA-CN to a mean of 0 and SD of 1 over 96 genotyping plates. Finally, quality control was performed by eliminating individuals with SD of autosomal probe intensities higher than 0.37. More details on the calculations and analysis pipeline used for mtDNA-CN can be found online (https://github.com/GrassmannLab/MT_UKB).

#### Outcome definition

Prevalent and incident diagnosis of type 2 diabetes was identified in UKB as the date of first appearance of non-insulin-dependent diabetes mellitus (data-field 130708 in the UKB database). This variable has been composed through a standard algorithm, performed by the UKB data management team, combining the data derived from hospital admissions (through linkage with the medical records from the National Health Service), general practitioners, death records and self-report. Detailed information of the algorithm and process can be found online (https://biobank.ndph.ox.ac.uk/showcase/ukb/docs/first_occurrences_outcomes.pdf). Based on the data of first appearance of type 2 diabetes and the data of enrolment information, we defined whether a case was prevalent (before enrolment) or incident (after enrolment).

#### Covariates

Covariates used in the study included genotyping batch, genetic principal component 1, genetic principal component 2, white blood cell counts, platelet counts, demographic measures (age, sex), anthropometric measures (BMI in kg/m^2^, waist circumference in cm, height in cm), self-reported lifestyles (use of cholesterol-lowering medication [Yes/No], smoking status [Never/Past/Current], physical activities [hours of being physically active per week]) and family (maternal + paternal) history of type 2 diabetes (Yes/No). Information on the covariates was collected at the time of study recruitment.

#### Power calculation

**Fig. 1:**
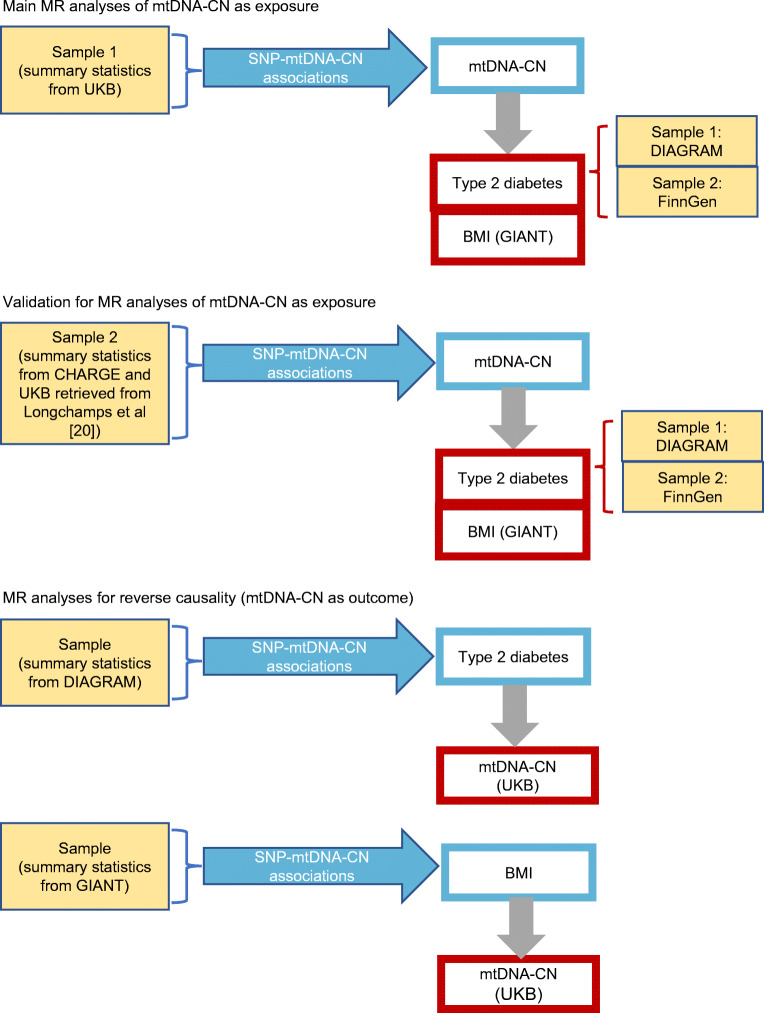
Study design diagram for bi-directional MR analyses. Grey arrows indicate MR effect estimates, traits in blue rectangles are exposures, traits in red rectangles are outcomes and GWAS data used for traits is presented in yellow rectangles

The statistical power in the MR analysis on type 2 diabetes was calculated using an online tool for binary outcome (https://shiny.cnsgenomics.com/mRnd/). Using data from the Diabetes Genetics Replication and Meta-analysis (DIAGRAM) consortium, we had sufficient power to detect a causal association with an OR of at least 1.07 per SD increase in blood mtDNA-CN using summary statistics either from UKB or Longchamps et al [20] with a significance level of 0.05 (Electronic supplementary material [ESM] Fig. 1).

#### Statistical analyses

Study characteristics for the population at baseline were expressed as mean with SD stratified by quartiles of blood mtDNA-CN. Missing values of variables were imputed using the package MICE which uses Bayesian polytomous regression for prediction of categorical values, predictive mean matching models for continuous missing values, and logistic regression models for prediction of binary missing values [21].

Participants without type 2 diabetes at baseline were followed until the occurrence of type 2 diabetes, mortality or loss to follow-up, whichever occurred first. Kaplan–Meier survival curves of blood mtDNA-CN in quartiles were provided. For the primary analysis, the association between blood mtDNA-CN as a continuous variable on type 2 diabetes was assessed using the Cox proportional hazard model. As secondary analyses, the analyses were repeated with blood mtDNA-CN stratified into quartiles. The 1st and 4th quartiles represent the lowest 25% and the highest 25% of blood mtDNA-CN, respectively. Three multivariable-adjusted Cox proportional hazard models were used to analyse the association between blood mtDNA-CN and type 2 diabetes: model 1 was adjusted for genotyping batch, the first two genetic principal components to correct for possible population stratification, white blood cell counts, platelet counts, age and sex; model 2 was additionally adjusted for BMI (in kg/m^2^); model 3 was additionally adjusted for use of cholesterol-lowering medication (Yes/No), smoking status (Never/Past/Current), physical activity (hours of being physically active per week), waist circumference (cm) and family history of type 2 diabetes (Yes/No).

The potential interactions between blood mtDNA-CN and sex and age were tested by adding interaction terms to the fully adjusted model. We also performed prespecified subgroup analyses stratified by sex and age (≤50 years, 50–60 years, ≥60 years).

In addition, multivariable-adjusted cross-sectional analyses were performed to investigate the association between blood mtDNA-CN and BMI by linear regression adjusted for genotyping batch, first two genetic principal components, white blood cell counts, platelet counts, age, sex, use of cholesterol-lowering medication, smoking status, physical activity and family history of type 2 diabetes. Also, similar models were applied to investigate the association between blood mtDNA-CN and whole-body lean mass, whole-body fat mass, liver enzymes including alanine aminotransferase (ALT), aspartate aminotransferase (AST), alkaline phosphatase (ALP) and gamma-glutamyl transferase (GGT). All variables except blood mtDNA-CN were log-transformed to approximate a normal distribution. The linear model of blood mtDNA-CN on whole-body lean mass was adjusted for genotyping batch, the first two genetic principal components, white blood cell counts, platelet counts, age, sex, height and fat mass. A similar model was used to investigate the association between blood mtDNA-CN and whole-body fat mass by replacing the fat mass with lean mass. The linear models of blood mtDNA-CN on liver enzymes were adjusted for genotyping batch, the first two genetic principal components, white blood cell counts, platelet counts, age, sex and glomerular filtration rate. The estimates derived from all linear models were exponentiated to assess the original effect of each variable per 1 SD increase in blood mtDNA-CN.

### Bi-directional Mendelian randomisation analyses

#### Exposure data source

A genome-wide association study (GWAS) for blood mtDNA-CN in the unrelated European population of the UKB was performed to provide a list of independent lead SNPs for use as instrumental variables for the exposure as well as the outcome set in the MR analyses for reverse causality. UKB genotyping and imputation data released in March 2018 was used to perform the GWAS (https://www.ukbiobank.ac.uk/media/cffi4mx5/ukb-genotyping-and-imputation-data-release-faq-v3-2-1.pdf). The analyses were done on the autosomal chromosomes adjusting for age, sex, white blood cell counts, platelet counts and the first ten genetic principal components. We excluded SNPs with a minor allele frequency <0.01 as well as SNPs with an imputation quality <0.3. SNPs with *p* values smaller than 5.0 × 10^−8^ were extracted and stored for the MR analyses. Instrumental variables were identified after the clumping process (window size = 10,000 kb, R^2^<0.001) which estimates linkage disequilibrium between SNPs by using the European population from the 1000 Genomes Project. GWAS in the present study were performed using linear mixed models implemented in the program BOLT_LMM (version 2.3.2) [22]. Visualisation of the results was performed using the R-based packages ggplot2 [23] and EasyStrata [24]. Variance in blood mtDNA-CN explained by genetic variants was calculated using equations that have been described previously [25].

In addition, 129 significant SNPs (*p*<5.0 × 10^−8^) associated with blood mtDNA-CN from Longchamps et al [20] were used as exposure in the validation analyses, which were provided in ESM Table 1. This GWAS study was performed in 465,809 European individuals from Cohorts for Heart and Aging Research in Genomic Epidemiology (CHARGE) and the UKB, adjusting for age, sex, principal components, DNA collection site, family structure and cell composition.

#### Outcome data source

Summary statistics of type 2 diabetes were extracted from two large databases, notably the DIAGRAM consortium (*N*=898,130) (available at: https://www.diagram-consortium.org/downloads.html) and the FinnGen study (*N*=215,654) (available at: https://finngen.gitbook.io/documentation/). The summary statistics of BMI were retrieved from the Genetic Investigation of Anthropometric Traits (GIANT) consortium (*N*=681,275), which is available on the Integrative Epidemiology Unit (IEU) GWAS database (available at: https://gwas.mrcieu.ac.uk/datasets/ieu-b-40/). More information on these data sources can be found in ESM Table 2.

#### MR analyses

Two-sample MR analyses were performed using the R-based statistical package TwoSampleMR (available at: http://github.com/MRCIEU/TwoSampleMR) [26]. This package is able to harmonise the effect sizes of SNPs for exposure and outcome, and connects to databases with thousands of complete GWAS summary data.

The study design for MR analyses is shown in Fig. 1. In the first set of two-sample MR analyses, we considered blood mtDNA-CN (UKB) (GWAS was performed in the present study) as exposure, and type 2 diabetes (DIAGRAM and FinnGen) and BMI (GIANT) as outcome. Analyses were repeated using data from the Longchamps et al as exposure [20]. The second set of MR analyses handled reverse causality taking type 2 diabetes (DIAGRAM) and BMI (GIANT) as exposure, and blood mtDNA-CN (UKB) from GWAS as outcome.

The primary MR analysis was conducted using the inverse variance weighted (IVW) method. The magnitudes of the causal effect were estimated by weighted regression of the average SNP-outcome effect on the average SNP-exposure effect with the intercept constrained to zero [27]. Given its assumption that all genetic instruments included are valid, this method could yield biased estimates in the presence of unbalanced horizontal pleiotropy, especially where the analysis contains many genetic instruments. Therefore, we also performed two additional sensitivity analyses by using other MR models, the weighted median estimator approach and the MR-Egger method. The weighted median estimator approach implemented the weighted median of the Wald ratios for all instrumental variables, which is able to tolerate up to (not including) 50% of the weight coming from invalid instrumental variables [28]. The MR-Egger method is sensitive to horizontal pleiotropy and the estimated value of intercept can be interpreted as an estimate of average pleiotropic effect across the genetic variants [29]. Results for MR analyses were reported in either OR for binary outcomes or estimates for continuous outcomes with accompanied 95% CI.

## Results

### Study population

The multivariable-adjusted analyses were conducted in a final sample of 285,967 individuals after excluding 7,207 individuals with type 2 diabetes at baseline. During the follow-up (median follow-up time 11.87 years; IQR 11.16–12.56 years), 15,111 individuals developed type 2 diabetes. The characteristics of participants stratified by quartile of blood mtDNA-CN after imputation are shown in Table 1, and the original baseline characteristics before imputation are shown in ESM Table 3. The mean age for the whole population was 56.8 (SD=8.00) years and 54.1% of participants were women.

**Table 1:** Baseline characteristics of the population by quartile of mtDNA-CN

Characteristic	Quartile 1 (*N*=71,492)	Quartile 2 (*N*=71,492)	Quartile 3 (*N*=71,491)	Quartile 4 (*N*=71,492)	Overall (*N*=285,967)
mtDNA-CN (SD)^a^	−1.12 (−5.094, −0.658)	−0.313 (−0.658, −0.004)	0.303 (−0.004, 0.656)	1.14 (0.656, 4.682)	−0.00423 (−5.094, 4.682)
Sex, female (%)	37587 (52.6)	38340 (53.6)	38911 (54.4)	39801 (55.7)	154639 (54.1)
Age (years)	57.4 (8.01)	56.9 (8.00)	56.6 (7.97)	56.1 (7.97)	56.8 (8.00)
White blood cell count (× 10^9^/l)	7.36 (1.83)	6.96 (1.72)	6.72 (1.70)	6.40 (2.50)	6.86 (1.99)
Platelet count (× 10^9^/l)	247 (57.8)	252 (57.8)	256 (59.0)	259 (62.9)	253 (59.6)
BMI (kg/m^2^)	27.6 (4.88)	27.4 (4.66)	27.2 (4.57)	26.9 (4.47)	27.3 (4.65)
Waist circumference (cm)	91.0 (13.6)	90.3 (13.3)	89.7 (13.1)	88.8 (12.9)	90.0 (13.2)
Whole-body fat mass (kg)	25.2 (9.80)	24.8 (9.41)	24.5 (9.26)	24.0 (9.05)	24.6 (9.39)
Whole-body lean mass (kg)	53.7 (11.5)	53.5 (11.5)	53.2 (11.4)	53.0 (11.4)	53.3 (11.5)
Physical activity (h/week)	26.4 (33.9)	27.0 (34.4)	26.9 (33.9)	27.1 (34.0)	26.8 (34.1)
Cholesterol-lowering medicine (%)	11941 (16.7)	11468 (16.0)	10974 (15.4)	10430 (14.6)	44813 (15.7)
Smoking status					
Never (%)	38362 (53.7)	39170 (54.8)	39715 (55.6)	40525 (56.7)	157772 (55.2)
Previous (%)	25027 (35.0)	24996 (35.0)	24806 (34.7)	24904 (34.8)	99733 (34.9)
Current (%)	8103 (11.3)	7326 (10.2)	6970 (9.7)	6063 (8.5)	28462 (10.0)
Family history of T2D (%)	11262 (15.8)	10959 (15.3)	11125 (15.6)	11156 (15.6)	44502 (15.6)
Follow-up time (years)	11.2 (2.30)	11.3 (2.12)	11.4 (2.06)	11.5 (1.99)	11.4 (2.13)
Height (cm)	169 (9.21)	169 (9.24)	169 (9.26)	169 (9.25)	169 (9.24)
ALT (U/l)	20.3 (3.10, 495)	20.2 (3.18, 490)	20.1 (3.25, 425)	19.7 (3.10, 491)	20.1 (3.10, 495)
ALP (U/l)	81.4 (8.00, 1360)	80.4 (14.8, 1420)	79.7 (14.2, 1360)	78.9 (14.1, 1420)	80.1 (8.00, 1420)
AST (U/l)	24.5 (5.10, 947)	24.4 (3.30, 711)	24.4 (5.10, 572)	24.3 (4.40, 584)	24.4 (3.30, 947)
GGT (U/l)	26.9 (5.50, 1170)	26.1 (5.10, 1120)	25.8 (5.00, 1160)	25.1 (5.20, 1160)	26.0 (5.00, 1170)

Continuous variables are presented as mean with SD or median with minimum and maximum values; categorial variables are presented as number with percentages

^a^The unit for blood mtDNA-CN is SD

T2D, type 2 diabetes

### Multivariable-adjusted analyses on incident type 2 diabetes

**Fig. 2:**
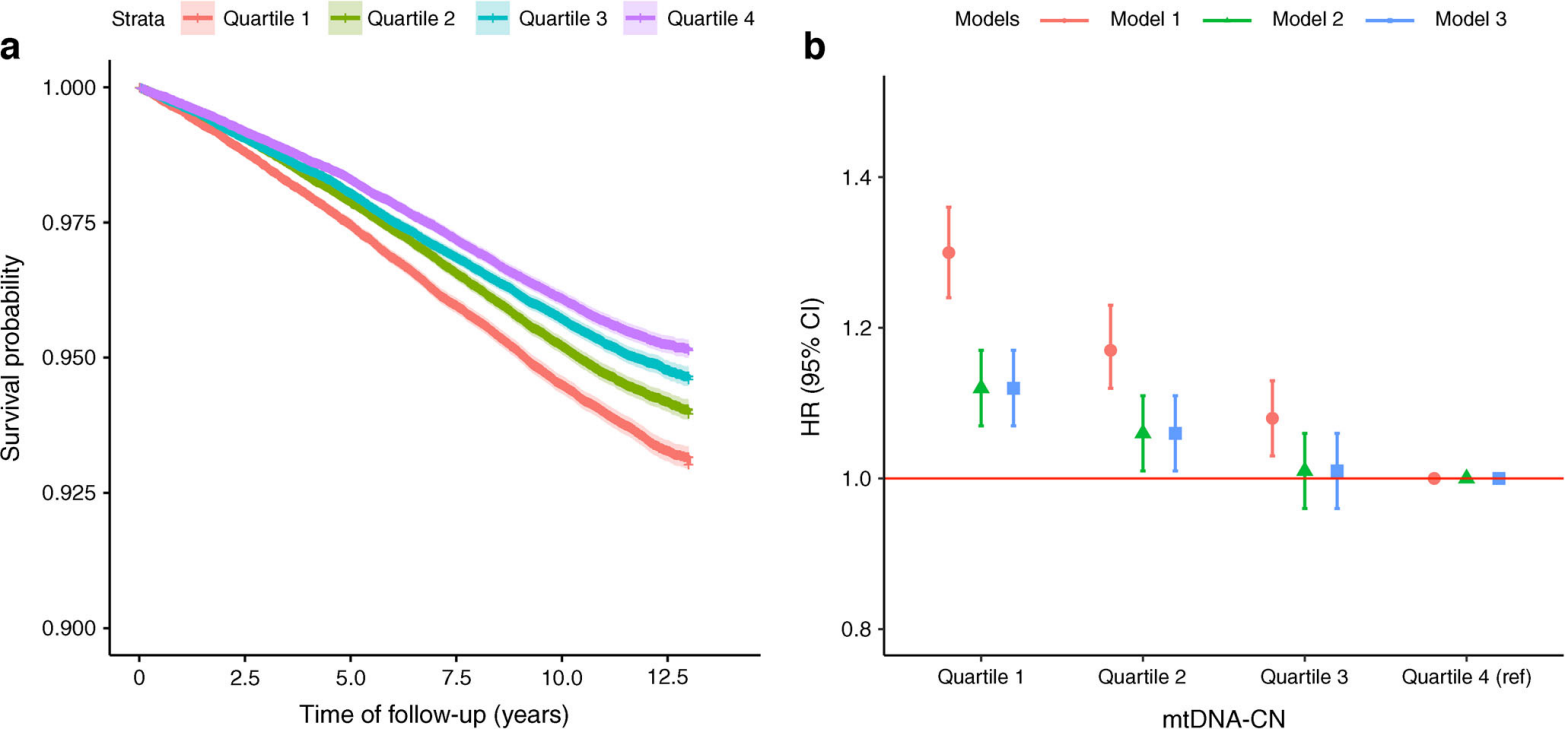
(**a**) Kaplan–Meier curve presenting time to develop incident type 2 diabetes by quartile of mtDNA-CN. The *x*-axis shows follow-up time in years and the *y*-axis shows the overall survival probability of developing type 2 diabetes. (**b**) HR for incident type 2 diabetes by quartiles of mtDNA-CN. Model 1 was adjusted for genotyping batch, principal component 1, principal component 2, white blood cell counts, platelet counts, age and sex; Model 2 was additionally adjusted for BMI based on model 1; Model 3 was additionally adjusted for BMI, medication use for cholesterol, smoking status, physical activities, waist circumference and family history of type 2 diabetes based on model 1

The Kaplan–Meier curve (Fig. 2a) illustrates that participants with a higher level of blood mtDNA-CN had a proportionally lower risk of incident type 2 diabetes.

The results from the multivariable-adjusted prospective analyses are presented in Fig. 2b and ESM Table 4. The multivariable-adjusted Cox proportional models show the higher level of blood mtDNA-CN is associated with lower risk of developing type 2 diabetes with HR of 0.90 (95% CI 0.89, 0.92) for model 1, 0.95 (95% CI 0.93, 0.97) for model 2 and 0.95 (95% CI 0.94, 0.97) for model 3 per 1 SD increase in blood mtDNA-CN. Similarly, taking the participants in the 4th quartile as reference group, model 1 shows participants in quartile 1 for blood mtDNA-CN had the highest risk of developing type 2 diabetes (HR 1.30 [95% CI 1.24, 1.36]); in addition, both quartile 2 and quartile 3 had higher risk of incident type 2 diabetes compared with the reference group (respectively, HR 1.17 [95% CI 1.12, 1.23], and HR 1.08 [95% CI 1.03, 1.13]). Results did somewhat attenuate after adjustment for BMI (model 2), and remained similar after further adjustment for other considered confounders (model 3).

We did not observe any interaction between blood mtDNA-CN with sex or age with *p* values for interaction of 0.06 and 0.61, respectively, as was also observed in subgroup analyses (ESM Table 5, ESM Table 6).

Higher blood mtDNA-CN was associated with lower BMI (−0.12 [95% CI −0.14, −0.10] kg/m^2^) in multivariable-adjusted cross-sectional analyses. The results from linear models of blood mtDNA-CN with whole-body lean mass, whole-body fat mass and liver enzymes are shown in ESM Table 7. Blood mtDNA-CN was not associated with whole-body lean mass, ALP, AST and ALT. A 1% decrease in whole-body fat mass and GGT were observed per SD increase in blood mtDNA-CN.

### Bi-directional MR analyses

In our newly conducted GWAS of blood mtDNA-CN in UKB, 3075 significant SNPs (*P*<5.0 × 10^−8^) were identified, of which 55 were independent lead SNPs (*R*^2^<0.001), which were used in the MR analyses as exposure (ESM Table 8). These independent lead SNPs explained 1.3% of variance in blood mtDNA-CN levels. The −log_10_(*p* value) plot and Q-Q plot for GWAS of blood mtDNA-CN are presented in ESM Fig. 2.

**Fig. 3:**
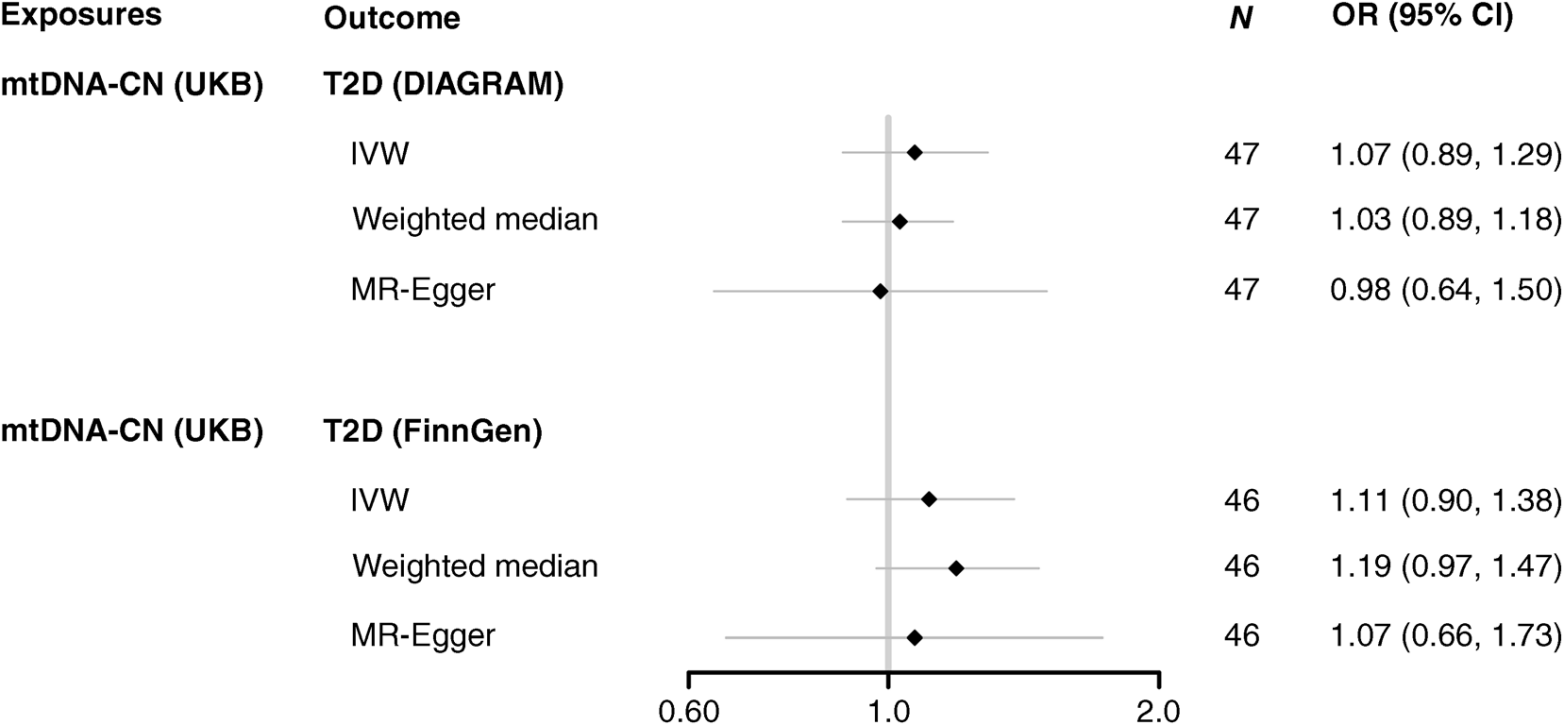
Forest plot of MR results for mtDNA-CN (UKB) and type 2 diabetes (DIAGRAM and FinnGen). *N* indicates the number of SNPs analysed in the MR analyses. OR indicates OR per 1 SD increase in mtDNA-CN. T2D, type 2 diabetes

**Fig. 4:**
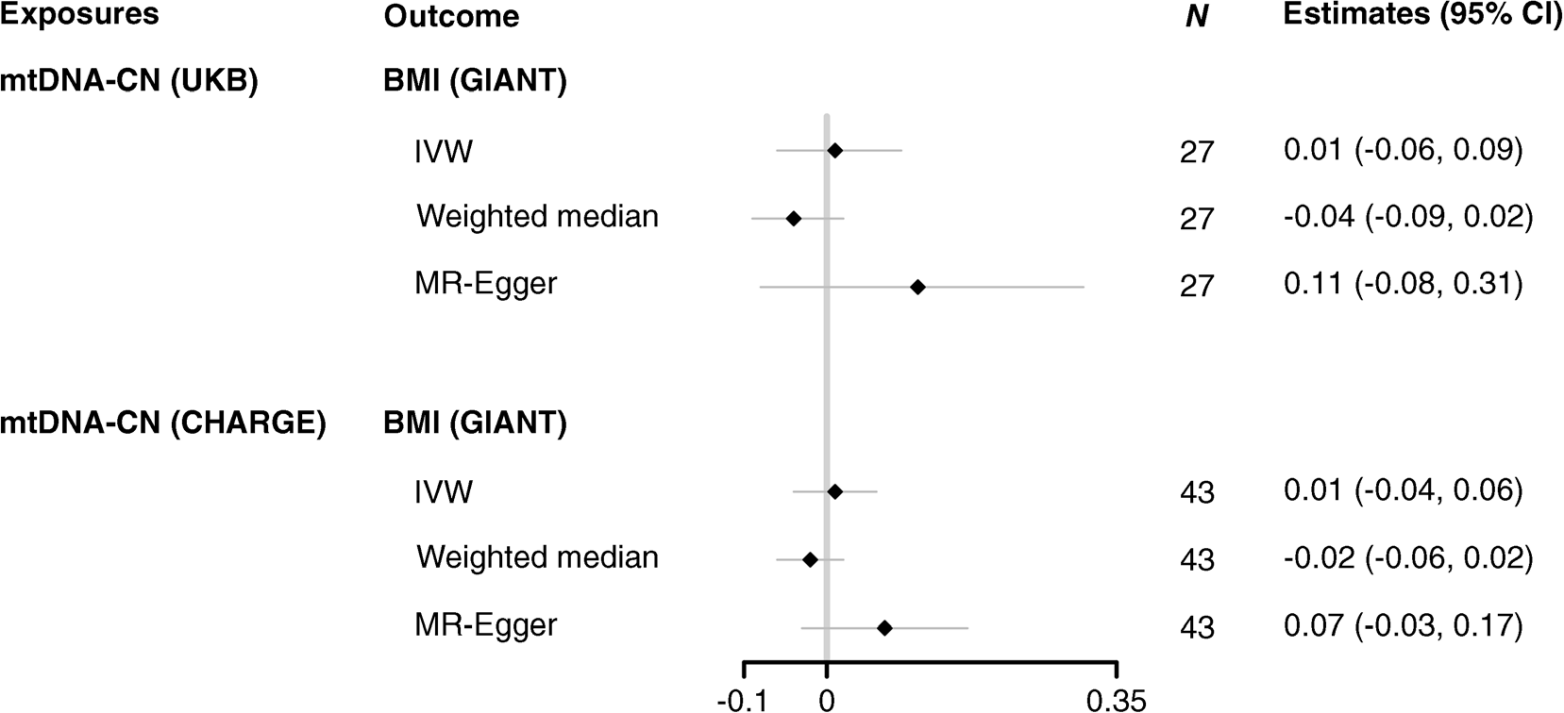
Forest plot of MR results for mtDNA-CN (UKB and CHARGE) and BMI (GIANT). Estimates are reported per 1 SD increase in mtDNA-CN. *N* indicates the number of SNPs analysed in the MR analyses

The results from the MR analyses taking 55 SNPs of blood mtDNA-CN from the UKB as exposure are shown in Fig. 3 and ESM Table 9. We observed that the OR per SD increase of blood mtDNA-CN on type 2 diabetes using the IVW method was 1.07 (95% CI 0.89, 1.29) for DIAGRAM as outcome data source and 1.11 (95% CI 0.90, 1.38) for FinnGen as outcome data source. Similar results were obtained when we used the previously identified SNPs from the larger Longchamps et al study [20] as exposure (ESM Fig. 3 and ESM Table 9) which explained 2.2% of variance in blood mtDNA-CN levels. The results from the MR analyses of blood mtDNA-CN on BMI using either UKB data or data from Longchamps et al [20] as exposure gave similar results indicating no evidence for a causal association between blood mtDNA-CN and BMI (Fig. 4 and ESM Table 10). The results from weighted median and MR-Egger were consistent with the IVW method in the above analyses.

**Fig. 5:**
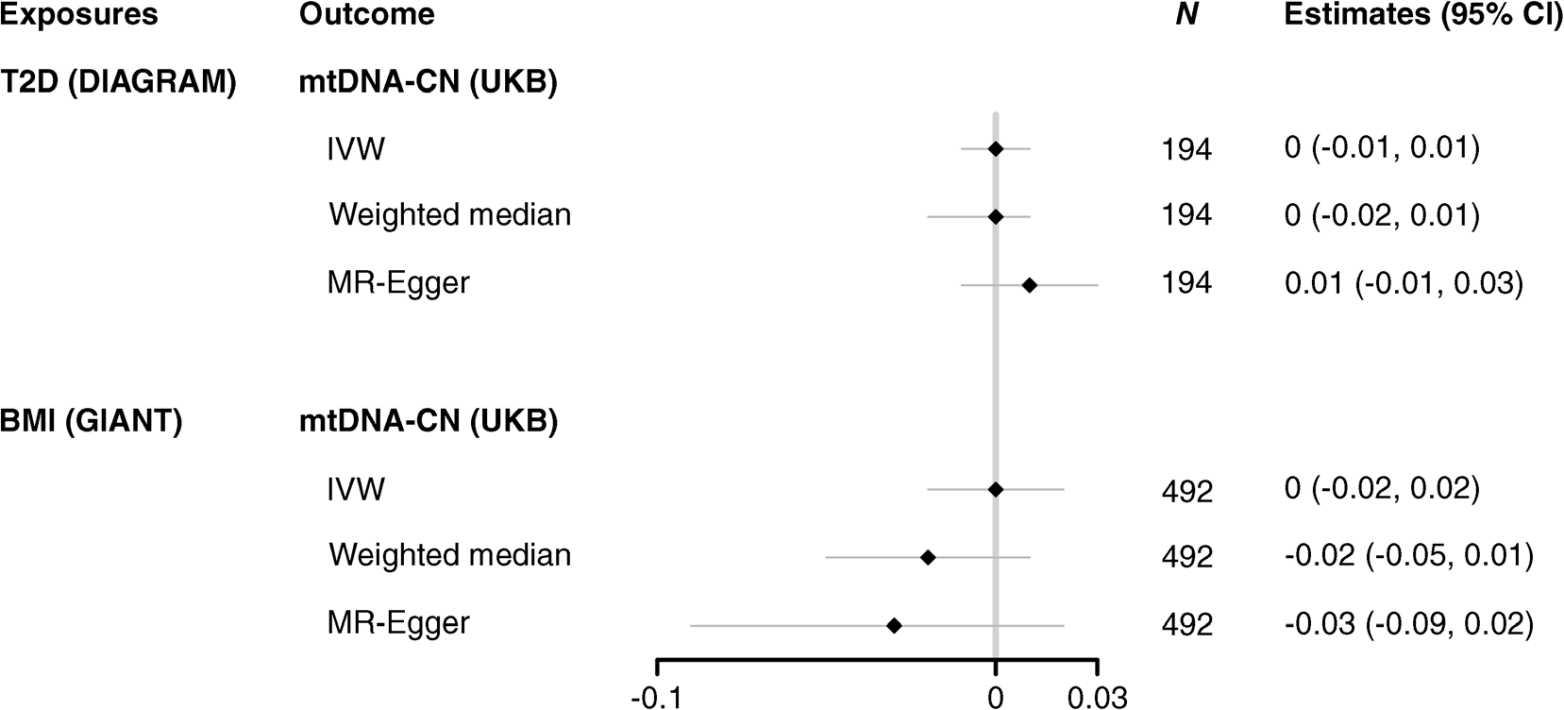
Forest plot of MR results for BMI (GIANT) and type 2 diabetes (DIAGRAM) associating with mtDNA-CN (UKB). Estimates were reported as having T2D vs not having T2D for MR analyses between T2D and mtDNA-CN. Estimates were reported as per 1 kg/m^2^ increase in BMI for MR analyses between BMI and mtDNA-CN. *N* indicates the number of SNPs analysed in the MR analyses. T2D, type 2 diabetes

The reverse MR was applied to investigate the potential causal association between genetically influenced BMI and type 2 diabetes on blood mtDNA-CN, respectively. Figure 5 illustrates the estimates of type 2 diabetes on blood mtDNA-CN and BMI on blood mtDNA-CN. For both exposures, we did not find evidence for a causal association, and consistent results were derived in the weighted median and MR-Egger analyses (ESM Table 11).

## Discussion

We found a lower level of blood mtDNA-CN to be associated with higher risk of incident type 2 diabetes. A weak association between blood mtDNA-CN and lower BMI was also found. However, no associations were observed between lower genetically influenced blood mtDNA-CN and higher type 2 diabetes risk nor higher BMI, suggesting that the observed associations are likely not causal. Also in the reverse direction, no evidence was found for a causal association of type 2 diabetes and of BMI with blood mtDNA-CN. Further regression analyses showed that blood mtDNA-CN was not associated with whole-body lean mass, AST, ALT and ALP, but was associated with whole-body fat mass and GGT, however, with very small observed effect sizes.

Three previous follow-up studies have investigated the association between blood mtDNA-CN and incident type 2 diabetes [13, 15, 16]. Our findings are in line with the results of Memon et al who found a lower level of blood mtDNA-CN to be associated with increased risk of incident type 2 diabetes in a follow-up study of 2387 middle-aged Swedish women [13]. However, research conducted in the Atherosclerosis Risk in Communities (ARIC) study found that mtDNA-CN in circulating leucocytes was not associated with risk of developing type 2 diabetes among 7713 white participants [15]. Similar results to the ARIC study were reported by Reiling et al using European participants from the Botnia and Rotterdam studies [16]. Our finding of an association of higher blood mtDNA-CN with lower BMI is similar to the findings of Liu et al [30] and Skuratovskaia et al [31]. The divergent results on type 2 diabetes may be due to the different characteristics of the populations, different covariates used in the Cox models, the data source of type 2 diabetes and the measurement methods for mtDNA-CN. However, the extremely large UKB cohort make it possible to observe the small effect sizes of the associations and the discrepancy may be explained by the differences in power.

We found no evidence, from the results of the MR analyses, for the hypothesis that low blood mtDNA-CN drives the risk of type 2 diabetes and obesity. This hypothesis is based on the assumption that blood mtDNA-CN reflects overall mitochondrial function including the mitochondrial function of tissues such as muscle and liver that play a role in type 2 diabetes. We further elaborated on this assumption by performing linear regression analyses between blood mtDNA-CN and whole-body lean and fat mass, and liver enzymes. We found that one SD increase in blood mtDNA-CN led to no change in lean mass, AST, ALT and ALP, and small decreases in GGT. This indicates that blood mtDNA-CN may not be a marker for mitochondrial function in muscle and a weak marker for mitochondrial function in liver. Although one study in monkeys reported that mitochondrial function in blood was associated with mitochondrial function in muscle [32], human trials have failed to show the correlation between mitochondrial respiration in peripheral blood and muscle in both women and men. This is in agreement with our finding that blood mtDNA-CN may not be a good marker for muscle mitochondrial function [33, 34]. The associations that have been observed between mtDNA-CN and liver disease are not consistent. Increased liver mtDNA-CN was found in patients with non-alcoholic fatty liver disease [35, 36], while decreased blood and liver mtDNA-CN was found in patients with biliary atresia [37, 38]. Although blood mtDNA-CN was associated with the liver enzyme GGT in our study, given the small effect sizes, the blood mtDNA-CN is a weak marker for mitochondrial functional in the liver.

Our data showed a clear observational association between blood mtDNA-CN and incident type 2 diabetes. Since the MR analyses did not find evidence for causality, it was expected that adjusting the observational association for BMI and other considered confounders would significantly attenuate the association. However, this was not the case, and only adjusting for BMI moderately attenuated the association. The most likely explanation is that the remaining association is due to residual confounding. In addition to unmeasured residual confounding, some measured variables may not be very precise. For example, the physical activities were collected based on the recall of participants, which may not be accurate.

Our study has some strengths and limitations. One of the strengths is that we included 285,967 unrelated European individuals without type 2 diabetes, which is a large sample size and large number (*N*=15,111) of incident type 2 diabetes cases. To our knowledge, this is the largest investigation on blood mtDNA-CN and type 2 diabetes. One of the limitations was that blood mtDNA-CN may not be a reflection of mitochondrial function in muscle and liver, which are relevant in type 2 diabetes pathogenesis, and therefore blood mtDNA-CN might not be a suitable marker for the involvement of these tissues in type 2 diabetes. Another limitation is that we only included European populations and the results cannot be extrapolated to other populations with different ancestries. In addition, there was some overlap in the data sources for exposure and outcome in the two-sample MR analyses. However, Minelli et al provide evidence that two-sample MR methods perform well in large one-sample MR studies even when the assumption of independence does not hold. Only results derived with the MR-Egger regression analysis should be interpreted with caution [39].

In conclusion, our results showed that blood mtDNA-CN was prospectively associated with incident type 2 diabetes, but MR analyses did not provide evidence for causality.

## Supplementary Information

ESM (PDF 476 kb)

## Data Availability

Data supporting the findings of this study have been deposited in the UK Biobank under project number 22474. Data are available on request after approval of a research proposal by UK Biobank resources and payment of an access fee.

## Abbreviations

ALP: Alkaline phosphatase
ALT: Alanine aminotransferase
ARIC: Atherosclerosis Risk in Communities
AST: Aspartate aminotransferase
CHARGE: Cohorts for Heart and Aging Research in Genomic Epidemiology
DIAGRAM: Diabetes Genetics Replication and Meta-analysis
GGT: Gamma-glutamyl transferase
GIANT: Genetic Investigation of Anthropometric Traits
GWAS: Genome-wide association study
IVW: Inverse variance weighted
L2R: Log_2_ transformed ratio
MR: Mendelian randomisation
mtDNA: Mitochondrial DNA
mtDNA-CN: Mitochondrial DNA copy number
UKB: UK Biobank
