# Supplementary material for: Assessment of the bi-directional relationship between blood mitochondrial DNA copy number and type 2 diabetes mellitus: a multivariable-adjusted regression and Mendelian randomisation study
Source: Diabetologia. 2022 Jul 22;65(10):1676–86. doi: 10.1007/s00125-022-05759-6 (PMC9477915; doi:10.1007/s00125-022-05759-6)
Supplement: Supplementary file 1 — (PDF 476 kb) [file 125_2022_5759_MOESM1_ESM.pdf]

Supplementary content

Association between mitochondrial DNA copy number and type 2 diabetes: a multivariable-adjusted regression and Mendelian Randomization study

Wenyi Wang*, Jiao Luo, Ko Willems van Dijk, Sara Hägg, Felix Grassmann, Leen M `t Hart, Diana van Heemst, Raymond Noordam

Table 1 Genetic instruments with significant threshold for mtDNA copy number retrieved from paper of Longchamps et al.

SNP	EA	Non-EA	EAF	Beta	SE	P
rs1569419	T	C	0,233191	-0,02295	0,002504	1,9E-19
rs3818157	G	A	0,538437	0,021182	0,00212	3E-24
rs204071	C	T	0,931713	-0,02249	0,004152	2,8E-08
1:156455314	CTT	C	0,31637	0,014484	0,002256	9,9E-11
rs2145380	G	C	0,352303	0,001937	0,002194	0,53
rs6425521	C	A	0,197495	-0,02174	0,002642	2,3E-17
rs9425311	G	T	0,509839	0,013351	0,0021	3,9E-10
1:205246482	TTTTG	T	0,614256	0,016314	0,002154	1,5E-13
rs10749636	G	A	0,238406	-0,01532	0,002477	1,1E-09
rs655029	G	A	0,290706	0,014052	0,002335	1,6E-09
rs711244	C	T	0,579539	0,013768	0,002122	1,8E-10
rs2302643	G	A	0,553774	-0,01212	0,002125	7,1E-09
rs151084028	T	G	0,993909	0,085386	0,01447	3,8E-09
rs865551	C	G	0,364543	0,015864	0,002189	3,5E-13
rs62641680	G	A	0,971137	0,107204	0,006253	1,5E-65
rs74874677	A	G	0,977259	0,099827	0,007026	6E-46
rs12052715	C	G	0,275224	0,016754	0,002343	3,3E-13
rs147820690	C	T	0,997407	-0,12394	0,020575	3,8E-09
rs78909033	G	A	0,866128	0,024616	0,003077	4,4E-15
rs13084580	C	T	0,886999	-0,02691	0,003309	4,2E-16
rs6786055	G	T	0,370122	0,014694	0,002175	2,7E-12
rs1354034	T	C	0,400257	0,031093	0,002136	3,4E-49
rs6778131	T	A	0,376044	0,012015	0,002176	2,3E-08
rs1420476	T	A	0,043704	-0,02322	0,005112	5,7E-06
rs34894010	C	G	0,957324	-0,03746	0,005212	1,6E-12
rs755492124	TAAAG	T	0,605827	-0,01375	0,002148	2,4E-11
rs6894574	T	C	0,333672	0,011287	0,002224	1,2E-07
rs2736100	C	A	0,501902	0,01726	0,002098	1,6E-16
rs34592828	G	A	0,955043	-0,03215	0,005056	5,8E-11
rs114694170	T	C	0,941548	-0,03657	0,004514	9,9E-16
rs56116444	T	G	0,924763	0,025006	0,00398	3,8E-10
rs193541	C	T	0,419551	-0,01303	0,002122	6,1E-10
rs926326	A	G	0,226937	-0,01712	0,002504	1,2E-11
rs2844484	A	G	0,385303	0,017398	0,002154	3,9E-16
rs45552734	C	T	0,880997	-0,02088	0,003239	7,3E-11
rs511515	A	G	0,299332	-0,02091	0,00229	5,2E-21
rs5745582	C	T	0,820528	-0,02559	0,002737	1,2E-21
rs4895441	A	G	0,726861	-0,01996	0,002355	9,5E-18
rs7744765	T	C	0,594993	0,015271	0,002137	3,1E-13
rs200957609	G	A	0,9976	-0,11682	0,021434	7,6E-08
rs35585318	T	C	0,606557	0,011286	0,002146	2,1E-07
rs6943701	A	T	0,823261	-0,01808	0,00275	2,5E-11

rs17260734	T	A	0,522974	-0,01124	0,002114	7,1E-08
rs445	C	T	0,903754	-0,02067	0,003546	3E-09
rs139141690	G	A	0,995243	0,098921	0,015597	2E-10
rs342293	C	G	0,541029	-0,03451	0,002101	7E-61
rs74750282	T	C	0,913187	-0,04784	0,003735	7,3E-37
rs602616	C	G	0,910591	0,019298	0,003701	3,2E-08
rs10085457	G	A	0,674259	-0,00919	0,002238	0,000034
rs3110823	A	C	0,830355	-0,03294	0,002793	5,9E-33
rs7800558	T	C	0,58247	0,012245	0,002126	1,2E-08
rs117728810	G	A	0,94297	0,023917	0,004571	8,7E-08
8:6701534	ACTC	A	0,498303	-0,01179	0,002104	1,9E-08
rs4284061	T	A	0,561259	0,020073	0,002187	6,3E-20
rs4841132	A	G	0,091291	0,027626	0,003654	6,1E-14
8:103218144	ATTGCTA TTATAAA TAAGCTT	A	0,935085	-0,02835	0,004272	1,3E-11
rs6986601	A	G	0,457191	0,011906	0,002103	7,7E-09
rs385893	T	C	0,477073	-0,01432	0,002099	1,2E-12
9:4838587	TAC	T	0,113928	-0,02069	0,003306	4,3E-10
rs7033052	G	C	0,46772	0,009051	0,002123	0,000017
rs12247015	A	G	0,581931	-0,04217	0,002126	4,4E-89
rs3876	C	T	0,923705	0,002929	0,003975	0,58
rs56356712	T	C	0,948209	-0,0097	0,004736	0,06
rs181771244	C	T	0,99401	-0,03816	0,014071	0,0033
rs7896518	A	G	0,572131	0,051452	0,00214	1,9E-127
rs73349121	G	C	0,98285	0,133878	0,008078	4,3E-61
rs7902510	C	T	0,790885	0,03186	0,002573	2,1E-36
rs11594179	C	T	0,764371	-0,02197	0,002473	1,5E-18
rs7080536	G	A	0,956708	0,032287	0,005223	6,3E-10
rs4910886	G	T	0,662039	-0,02258	0,002217	6,2E-25
rs2241942	G	A	0,758987	0,013837	0,002463	1,7E-08
rs11235573	T	C	0,448284	0,011221	0,00211	3,8E-08
rs74472890	T	C	0,950634	0,026591	0,00484	1,2E-08
rs1362214	A	G	0,518908	-0,0242	0,002113	8,5E-31
rs1127787	G	A	0,831123	0,018808	0,002796	3,5E-11
rs2015599	G	A	0,540794	-0,01207	0,002104	1,5E-08
rs6580981	G	A	0,540754	-0,01423	0,002115	2,4E-11
rs1716505	C	G	0,683007	0,01328	0,002285	4,7E-09
rs12426673	G	T	0,417326	0,014413	0,002127	2,2E-11
rs749140768	AGGCAC CTCTTCA CAGGAC	A	0,919829	-0,0289	0,003899	1,1E-14
rs11553699	A	G	0,864607	-0,05075	0,003194	1,2E-57
rs7987027	T	C	0,459373	-0,0111	0,002103	1,2E-07
rs1760940	A	C	0,75281	-0,0271	0,00243	2E-29
rs2771358	T	C	0,745471	0,011995	0,002405	2,4E-07
rs17477725	C	G	0,414854	0,009683	0,002141	6,9E-06

rs4427713	T	C	0,446147	0,011989	0,002118	5,9E-09
rs117948349	G	A	0,966604	0,034522	0,005863	2,5E-09
rs59488041	T	A	0,864626	0,0238	0,003071	2,5E-14
rs261290	T	C	0,346067	-0,01257	0,002209	1,8E-08
rs141227171	G	C	0,997486	0,092557	0,023313	0,00011
rs3087374	C	A	0,918931	-0,02366	0,003836	2,6E-10
rs151234	G	C	0,869485	0,020078	0,003118	7,8E-11
rs289713	T	A	0,189079	0,01878	0,002696	2,5E-12
rs55823018	C	T	0,675508	0,013095	0,002253	5,9E-09
rs7213347	G	C	0,301487	0,013164	0,002284	2E-09
rs12451698	A	G	0,759979	0,019454	0,002456	9,9E-16
rs12601687	G	A	0,890455	-0,03014	0,003368	2,2E-19
rs1967556	T	G	0,473554	-0,02287	0,002107	1,3E-27
rs17850455	C	G	0,987947	-0,12051	0,010246	5,2E-33
rs11867543	C	T	0,862391	-0,01846	0,003046	5,5E-10
rs680478	C	T	0,246875	0,016511	0,002435	7,7E-12
rs77261872	C	T	0,87456	-0,03152	0,003178	4,5E-23
rs17758695	C	T	0,970637	0,042608	0,006211	2E-12
rs28665408	A	C	0,433817	-0,02023	0,00212	4,7E-21
rs12955015	C	A	0,021241	0,040373	0,007298	4,3E-08
rs10411696	T	G	0,527576	0,011692	0,002102	2,3E-08
rs11085147	C	T	0,903017	-0,09148	0,003632	3E-141
rs3218221	G	A	0,995578	0,096118	0,016601	3,7E-09
rs142158911	G	A	0,884207	-0,01751	0,003301	4,6E-08
rs57843631	C	T	0,980306	-0,05539	0,007818	2,4E-12
rs139891465	C	T	0,957814	-0,04889	0,005282	1,3E-20
rs10419397	G	A	0,70588	0,032272	0,002301	7,1E-46
rs35586766	G	A	0,905494	-0,03759	0,003595	4,5E-26
19:19756073	AGCC	A	0,92707	-0,02342	0,004125	1,1E-08
rs7412	C	T	0,919735	-0,04013	0,003861	1,3E-24
rs11667430	A	G	0,603106	-0,00857	0,002168	0,00011
rs1613662	G	A	0,165549	0,019946	0,002821	5,4E-13
rs11668201	A	T	0,81049	0,015043	0,00269	9,6E-09
rs11696739	G	A	0,622051	0,018931	0,002161	7,9E-19
rs156355	T	C	0,536062	-0,02446	0,002158	1,6E-30
rs4814776	C	A	0,670582	0,036218	0,002236	5,4E-59
rs185387034	A	G	0,990547	0,063137	0,011686	4E-08
rs754169	T	A	0,463532	-0,02735	0,0021	1,2E-38
rs76599088	C	T	0,983417	-0,07962	0,008206	1,9E-22
rs2426092	A	C	0,548316	-0,01235	0,002109	3,9E-09
rs577050795	C	CA	0,524868	-0,01087	0,002104	2,1E-07
rs2245947	G	T	0,322015	-0,03896	0,002245	2,5E-68
rs75107793	G	A	0,927893	-0,03018	0,004083	2,3E-13
rs12148	T	G	0,388911	0,016642	0,002151	5,7E-15

SNP: single-nucleotide polymorphisms; EA: effect allele; Non-EA: Non effect allele; EAF: effect allele frequency; SE: standard error.

Table 2: GWAS data source for Mendelian Randomization

**Phenotype**	**Consortium**	**Population**	**Cases/ Controls**
Type 2 diabetes (*Mahajan A, et al, 2018, Nature Genetics*)	DIAGRAM	European ancestry	74124/ 824006
Type 2 diabetes (*Data freeze 5, release in 2021*)	FinnGen	Finnish	32469/ 183185
BMI (*Yengo L, et al, 2018, Hum Mol Genet.)*	GIANT	European ancestry	681275 (total)

DIAGRAM: Diabetes Genetics Replication and Meta-analysis consortium; GIANT: The Genetic Investigation of Anthropometric Traits consortium.

Table 3 Baseline characteristics of the population by quartile of mtDNA copy number before imputation

	**Quartile 1 (N=71492)**	**Quartile 2 (N=71492)**	**Quartile 3 (N=71491)**	**Quartile 4 (N=71492)**	**Overall (N=285967)**
**Sex**					
Female	37587 (52.6%)	38340 (53.6%)	38911 (54.4%)	39801 (55.7%)	154639 (54.1%)
Male	33905 (47.4%)	33152 (46.4%)	32580 (45.6%)	31691 (44.3%)	131328 (45.9%)
**Age**					
Mean (SD)	57.4 (8.01)	56.9 (8.00)	56.6 (7.97)	56.1 (7.97)	56.8 (8.00)
**White blood cell count**					
Mean (SD)	7.36 (1.82)	6.96 (1.71)	6.72 (1.67)	6.40 (2.52)	6.86 (1.99)
Missing	2148 (3.0%)	2121 (3.0%)	2071 (2.9%)	2169 (3.0%)	8509 (3.0%)
**Platelet count**					
Mean (SD)	247 (57.7)	252 (57.9)	256 (58.9)	259 (63.0)	253 (59.6)
Missing	2148 (3.0%)	2119 (3.0%)	2069 (2.9%)	2169 (3.0%)	8505 (3.0%)
**BMI**					
Mean (SD)	27.6 (4.89)	27.4 (4.66)	27.2 (4.57)	26.9 (4.47)	27.3 (4.65)
Missing	253 (0.4%)	226 (0.3%)	196 (0.3%)	194 (0.3%)	869 (0.3%)
**Waist circumference**					
Mean (SD)	91.0 (13.6)	90.3 (13.3)	89.7 (13.1)	88.8 (12.9)	90.0 (13.2)
Missing	134 (0.2%)	115 (0.2%)	114 (0.2%)	98 (0.1%)	461 (0.2%)
**Whole-body fat mass**					
Mean (SD)	25.2 (9.74)	24.8 (9.36)	24.5 (9.22)	24.0 (9.00)	24.6 (9.34)
Missing	1463 (2.0%)	1348 (1.9%)	1214 (1.7%)	1217 (1.7%)	5242 (1.8%)
**Whole-body lean mass**					
Mean (SD)	53.7 (11.5)	53.5 (11.5)	53.3 (11.5)	53.0 (11.4)	53.4 (11.5)

	Quartile 1 (N=71492)	Quartile 2 (N=71492)	Quartile 3 (N=71491)	Quartile 4 (N=71492)	Overall (N=285967)
Missing	1334 (1.9%)	1237 (1.7%)	1116 (1.6%)	1093 (1.5%)	4780 (1.7%)
**Physical activity**					
Mean (SD)	26.5 (33.9)	27.1 (34.6)	27.1 (34.0)	27.2 (34.1)	27.0 (34.1)
Missing	14029 (19.6%)	13419 (18.8%)	13257 (18.5%)	13088 (18.3%)	53793 (18.8%)
**Cholesterol-lowering**					
No	59551 (83.3%)	60024 (84.0%)	60517 (84.6%)	61062 (85.4%)	241154 (84.3%)
Yes	11941 (16.7%)	11468 (16.0%)	10974 (15.4%)	10430 (14.6%)	44813 (15.7%)
**Smoking status**					
Never	38211 (53.4%)	39055 (54.6%)	39572 (55.4%)	40413 (56.5%)	157251 (55.0%)
Previous	24922 (34.9%)	24915 (34.9%)	24729 (34.6%)	24815 (34.7%)	99381 (34.8%)
Current	8077 (11.3%)	7299 (10.2%)	6949 (9.7%)	6047 (8.5%)	28372 (9.9%)
Missing	282 (0.4%)	223 (0.3%)	241 (0.3%)	217 (0.3%)	963 (0.3%)
**Family hitory of T2D**					
No	60230 (84.2%)	60533 (84.7%)	60366 (84.4%)	60336 (84.4%)	241465 (84.4%)
Yes	11262 (15.8%)	10959 (15.3%)	11125 (15.6%)	11156 (15.6%)	44502 (15.6%)
**Follow-up time**					
Mean (SD)	11.2 (2.30)	11.3 (2.12)	11.4 (2.06)	11.5 (1.99)	11.4 (2.13)
**Height**					
Mean (SD)	169 (9.22)	169 (9.24)	169 (9.26)	169 (9.25)	169 (9.24)
Missing	167 (0.2%)	153 (0.2%)	135 (0.2%)	126 (0.2%)	581 (0.2%)
**ALT**					
Median [Min, Max]	20.3 [3.35, 495]	20.2 [3.18, 440]	20.1 [3.25, 425]	19.7 [3.10, 491]	20.1 [3.10, 495]
Missing	3416 (4.8%)	3339 (4.7%)	3380 (4.7%)	3273 (4.6%)	13408 (4.7%)
**ALP**					

	Quartile 1 (N=71492)	Quartile 2 (N=71492)	Quartile 3 (N=71491)	Quartile 4 (N=71492)	Overall (N=285967)
Median [Min, Max]	81.5 [8.00, 1360]	80.4 [14.8, 1270]	79.7 [14.2, 970]	78.9 [14.1, 1420]	80.1 [8.00, 1420]
Missing	3382 (4.7%)	3310 (4.6%)	3366 (4.7%)	3246 (4.5%)	13304 (4.7%)
**AST**					
Median [Min, Max]	24.5 [5.10, 947]	24.4 [3.30, 711]	24.4 [6.00, 572]	24.3 [4.40, 584]	24.4 [3.30, 947]
Missing	3647 (5.1%)	3538 (4.9%)	3599 (5.0%)	3494 (4.9%)	14278 (5.0%)
**GGT**					
Median [Min, Max]	26.9 [5.60, 1170]	26.2 [5.10, 1120]	25.7 [5.00, 1160]	25.2 [5.20, 1160]	26.0 [5.00, 1170]
Missing	3421 (4.8%)	3349 (4.7%)	3395 (4.7%)	3286 (4.6%)	13451 (4.7%)

Missing values of parameters were imputed using package MICE which used Bayesian polytomous regression for prediction of categorical values, linear regression models for continuous missing values and logistic regression model for prediction of binary missing values. The unit for all variables could be found in Table 1. BMI: body mass index; T2D: type 2 diabetes; ALT: alanine aminotransferase; AST: aspartate aminotransferase; ALP: alkaline phosphatase; GGT: gamma glutamyl transferase.

Table 4: Hazard ratio of incident type 2 diabetes by quartile of mtDNA copy number

	Model	HR	LowerCI	UpperCI
**Quartile 1**	Model 1	1.30	1.24	1.36
**Quartile 2**		1.17	1.12	1.23
**Quartile 3**		1.08	1.03	1.13
**Quartile 4**		Reference	Reference	Reference
**Quartile 1**	Model 2	1.13	1.08	1.18
**Quartile 2**		1.07	1.02	1.12
**Quartile 3**		1.02	0.97	1.97
**Quartile 4**		Reference	Reference	Reference
**Quartile 1**	Model 3	1.12	1.07	1.17
**Quartile 2**		1.06	1.01	1.11
**Quartile 3**		1.01	0.96	1.06
**Quartile 4**		Reference	Reference	Reference
**Continuous**	Model 1	0.90	0.89	0.92
**Continuous**	Model 2	0.95	0.93	0.97
**Continuous**	Model 3	0.95	0.94	0.97

Model 1 was adjusted for genotyping batch, principal component 1, principal component 2, white blood cell counts, platelet counts, age and sex; model 2 was additionally adjusted for BMI based on model 1; model 3 was additionally adjusted for BMI, medication use for cholesterol, smoking status, physical activities, waist circumference and family history of type 2 diabetes based on model 1. CI: confidence interval.

Table 5: Hazard ratio of incident type 2 diabetes stratified by sex

	N (case/control)	HR	LowerCI	UpperCI
**Male**				
Model 1	131328	0.92	0.90	0.94
Model 2	(8974/122354)	0.96	0.94	0.98
Model 3		0.97	0.95	0.99
**Female**				
Model 1	154639	0.88	0.86	0.90
Model 2	(6137/148502)	0.94	0.92	0.96
Model 3		0.94	0.92	0.96

Model 1 was adjusted for genotyping batch, principal component 1, principal component 2, white blood cell counts, platelet counts, age and sex; model 2 was additionally adjusted for BMI based on model 1; model 3 was additionally adjusted for BMI, medication use for cholesterol, smoking status, physical activities, waist circumference and family history of type 2 diabetes based on model 1. CI: confidence interval.

Table 6: Hazard ratio of incident type 2 diabetes stratified by age

	N (case/control)	HR	LowerCI	UpperCI
**<=50 years**				
Model 1	71897	0.90	0.86	0.94
Model 2	(1930/69967)	0.96	0.92	1.01
Model 3		0.96	0.92	1.01
**50~60 years**				
Model 1	86776	0.88	0.86	0.91
Model 2	(4130/82646)	0.94	0.91	0.97
Model 3		0.95	0.92	0.98
**>=60 years**				
Model 1	127294	0.91	0.89	0.93
Model 2	(9051/118243)	0.95	0.93	0.97
Model 3		0.95	0.93	0.97

Model 1 was adjusted for genotyping batch, principal component 1, principal component 2, white blood cell counts, platelet counts, age and sex; model 2 was additionally adjusted for BMI based on model 1; model 3 was additionally adjusted for BMI, medication use for cholesterol, smoking status, physical activities, waist circumference and family history of type 2 diabetes based on model 1. CI: confidence interval.

Table 7: Results of follow-up study using linear regression model

Dependent variables	Independent variables	Covariates	Estimates	95% CI
Whole-body lean mass	mtDNA-CN	Adjusting for genotyping batch, principal component 1, principal component 2, white blood cell counts, platelet counts, age, sex, height and fat mass	1.00	1.00-1.00
Whole-body fat mass	mtDNA-CN	Adjusting for genotyping batch, principal component 1, principal component 2, white blood cell counts, platelet counts, age, sex, height and lean mass	0.99	0.98-0.99
ALT	mtDNA-CN		1.00	1.00-1.00
AST	mtDNA-CN	Adjusted for genotyping batch, principal component 1, principal component 2, white blood cell counts, platelet counts, age and sex	1.00	1.00-1.00
ALP	mtDNA-CN		1.00	1.00-1.00
GGT	mtDNA-CN		0.99	0.98-0.99

*All parameters except mtDNA-CN were firstly logarithm transformed to achieve approximate normality and then applied in the linear regression models. The estimates shown were exponentiated to get original effect of each parameter per 1 SD increase of mtDNA-CN. The unit for whole-body lean mass and whole-body fat mass is kg and the unit for ALT, AST, ALP and GGT is U/L. ALT: alanine aminotransferase; AST: aspartate aminotransferase; ALP: alkaline phosphatase; GGT: gamma glutamyl transferase.

Table 8: Independent lead SNPs of mtDNA copy number derived from GWAS of mtDNA conducted in the present paper

SNP	EA	Non-EA	eaf	beta	SE	pval
rs10900604	A	G	0,795089	0,0181271	0,00322354	1,9E-08
rs528000664	T	C	0,534825	0,0158269	0,00262699	1,7E-09
rs1172125	A	T	0,374367	-0,0150623	0,00265943	1,5E-08
rs62641680	G	A	0,970353	0,0785548	0,00756122	2,8E-25
rs74874677	A	G	0,977202	0,0700895	0,00858639	3,3E-16
rs2729705	T	C	0,417226	0,0147205	0,00260015	1,5E-08
rs1354034	T	C	0,399053	0,0239568	0,00262674	7,4E-20
rs71298382	A	G	0,972821	-0,0453732	0,00829349	4,5E-08
rs111623827	C	T	0,58424	0,0149236	0,00273153	4,7E-08
rs518867	T	C	0,376174	-0,0220382	0,00264624	8,2E-17
rs114694170	T	C	0,940441	-0,033356	0,00548359	1,2E-09
rs3846730	C	T	0,761247	0,0190545	0,00301087	2,5E-10
rs9267516	C	T	0,636789	0,0155697	0,0027684	1,9E-08
rs210143	T	C	0,299126	-0,0234445	0,0028084	6,9E-17
rs9361858	T	G	0,581182	-0,0146758	0,00261708	0,00000002
rs4895441	A	G	0,725837	-0,0167342	0,00288423	6,5E-09
rs2158799	C	G	0,39193	0,0151341	0,00264212	0,00000001
rs56388170	G	T	0,708692	0,0219034	0,00284616	1,4E-14
rs1761673	C	T	0,530305	0,0154835	0,00259505	2,4E-09
rs42031	A	T	0,791783	0,019553	0,00316992	6,9E-10
rs445	C	T	0,906334	-0,0336523	0,00440482	2,2E-14
rs342293	C	G	0,540028	-0,0307569	0,00257745	7,9E-33
rs77236693	C	T	0,90395	-0,0272389	0,00438975	5,4E-10
rs6467603	A	T	0,18446	0,0273222	0,00339381	8,2E-16
rs4841132	A	G	0,0923516	0,0306982	0,00444507	5E-12
rs1037699	C	T	0,922533	-0,0266273	0,00481048	3,1E-08
rs59697075	C	T	0,412976	-0,0157101	0,0026331	2,4E-09
rs4390300	G	A	0,535177	-0,0331528	0,00258208	9,7E-38
rs7080386	C	A	0,58535	0,0372155	0,00260885	3,6E-46
rs73349121	G	C	0,982755	0,107112	0,00986125	1,7E-27
rs741738	G	A	0,813505	0,0188346	0,00330894	1,3E-08
rs4910887	T	G	0,663382	-0,0176052	0,00271634	9,1E-11
rs61908718	C	T	0,349634	-0,0162801	0,00269966	1,6E-09
rs12146715	A	C	0,830945	0,0187388	0,00342165	4,3E-08
rs10775004	G	A	0,425073	0,0154489	0,0026452	5,2E-09
rs597808	A	G	0,483575	0,0163736	0,00258197	2,3E-10
rs11553699	A	G	0,864055	-0,0408627	0,00390833	1,4E-25
rs12147688	G	T	0,742866	-0,0279677	0,00295272	2,7E-21
rs59488041	T	A	0,864988	0,0224481	0,00375905	2,3E-09
rs3816117	T	C	0,513483	0,0141104	0,00256637	3,8E-08
rs2063185	T	C	0,284733	0,0155128	0,00284554	0,00000005
rs9909104	T	C	0,755067	0,0183319	0,00298103	7,7E-10

rs1967556	T	G	0,473289	-0,0143608	0,00258189	2,7E-08
rs8066846	T	C	0,420525	-0,0284156	0,00261278	1,5E-27
rs17850455	C	G	0,988375	-0,123468	0,0127913	4,8E-22
rs16978036	G	T	0,872463	-0,0234647	0,00384303	1E-09
rs1790961	T	G	0,475357	-0,0195996	0,00256884	2,3E-14
rs11085147	C	T	0,90095	-0,0773809	0,00440197	3,5E-69
rs56069439	C	A	0,704179	0,0284025	0,00280967	5E-24
rs1065853	G	T	0,919307	-0,0349925	0,00471231	1,1E-13
rs1613662	G	A	0,166306	0,0232342	0,00344424	1,5E-11
rs156355	T	C	0,539311	-0,0233435	0,00264143	9,7E-19
rs6136489	T	G	0,66756	0,0348107	0,00272242	1,9E-37
rs11697739	T	C	0,46513	-0,0264395	0,00256813	7,3E-25
rs2245946	G	A	0,320106	-0,0317227	0,00274979	8,6E-31

SNP: single-nucleotide polymorphisms; EA: effect allele; Non-EA: Non effect allele; EAF: effect allele frequency; SE: standard error.

Table 9 Mendelian Randomization results of mtDNA copy number on the risk of type 2 diabetes

Expsoure	Outcome	N	Odds Ratio	LowerCI	UpperCI
mtDNA-CN (UKB)	T2D (DIAGRAM)				
	IVW	47	1.07	0.89	1.29
	weighted median	47	1.03	0.89	1.18
	MR Egger	47	0.98	0.64	1.50
	T2D (FinnGen)				
	IVW	46	1.11	0.90	1.38
	weighted median	46	1.19	0.97	1.47
	MR Egger	46	1.07	0.66	1.73
mtDNA-CN (CHARGE)	T2D (DIAGRAM)				
	IVW	76	1.04	0.90	1.20
	weighted median	76	1.02	0.91	1.16
	MR Egger	76	0.91	0.70	1.20
	T2D (FinnGen)				
	IVW	73	0.99	0.83	1.17
	weighted median	73	0.96	0.80	1.14
	MR Egger	73	0.94	0.68	1.29

Table 10 Mendelian Randomization results of mtDNA copy number on the risk of body mass index

Exposure	outcome	N	Estimates	LowerCI	UpperCI
mtDNA-CN (UKB)	BMI (GIANT)				
	IVW	27	0.01	-0.06	0.09
	weighted median	27	-0.04	-0.09	0.02
	MR Egger	27	0.11	-0.08	0.31
mtDNA-CN (CHARGE)	BMI (GIANT)				
	IVW	43	0.01	-0.04	0.06
	weighted median	43	-0.02	-0.06	0.02
	MR Egger	43	0.07	-0.03	0.17

Table 11 Mendelian Randomization results of type 2 diabetes and BMI on mtDNA copy number

Exposure	Outcome	N	Estimates	LowerCI	UpperCI
T2D (DIAGRAM)	mtDNA-CN (UKB)				
	IVW	194	0	-0.01	0.01
	weighted median	194	0	-0.02	0.01
	MR Egger	194	0.01	-0.01	0.03
BMI (GIANT)	mtDNA-CN (UKB)				
	IVW	492	0	-0.02	0.02
	weighted median	492	-0.02	-0.05	0.01
	MR Egger	492	-0.03	-0.09	0.02

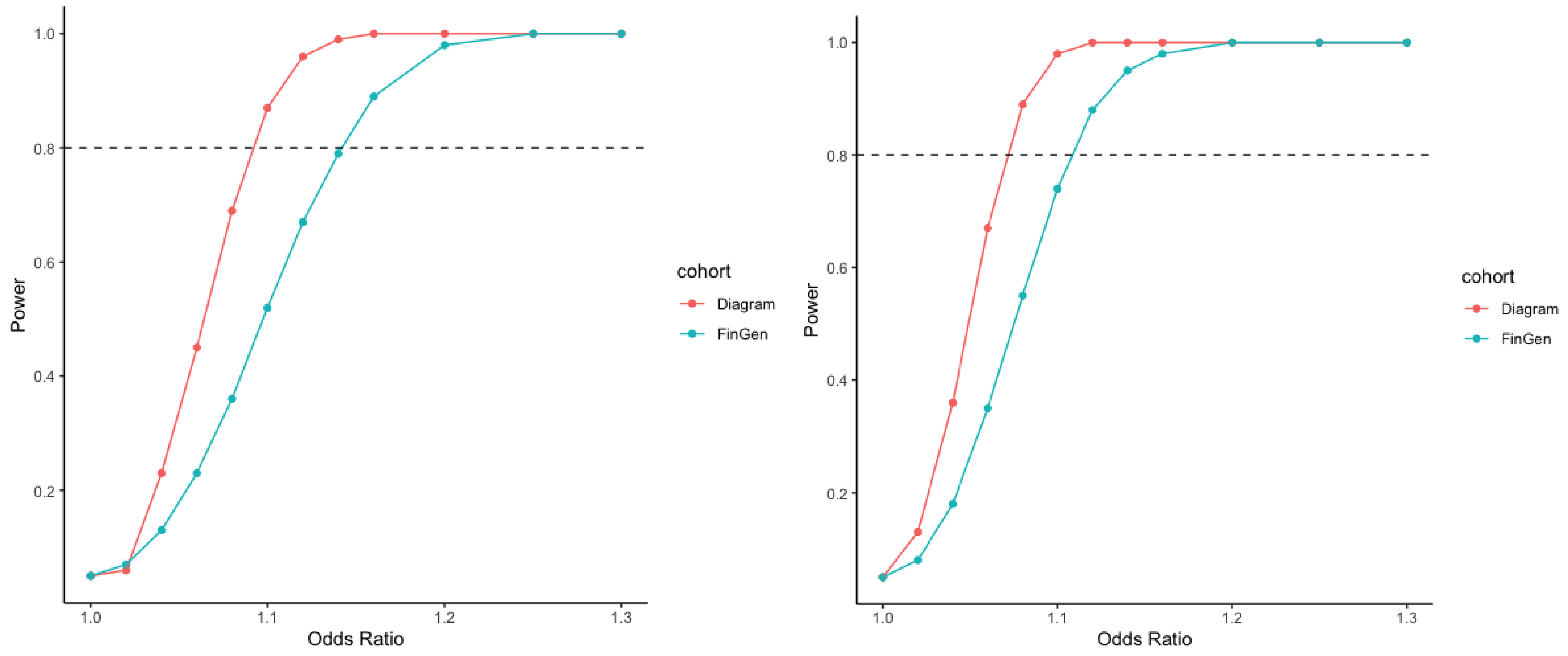

Figure 1 Statistical power of Mendelian Randomization analyses. The left figure was calculated with variance in blood mtDNA-CN explained by genetic variants derived from UKB (R^2=1.3%). The right figure was calculated with variance in blood mtDNA-CN explained by genetic variants derived from Longchamp et. al. paper (R^2=2.2%). Dashed black line indicates statistical power of 0.8. In the calculation, the significance level was set to be 0.05 and the true odds ratio of the outcome variable per standard deviation of the exposure variable was set to be ranging from 1.0 to 1.3.

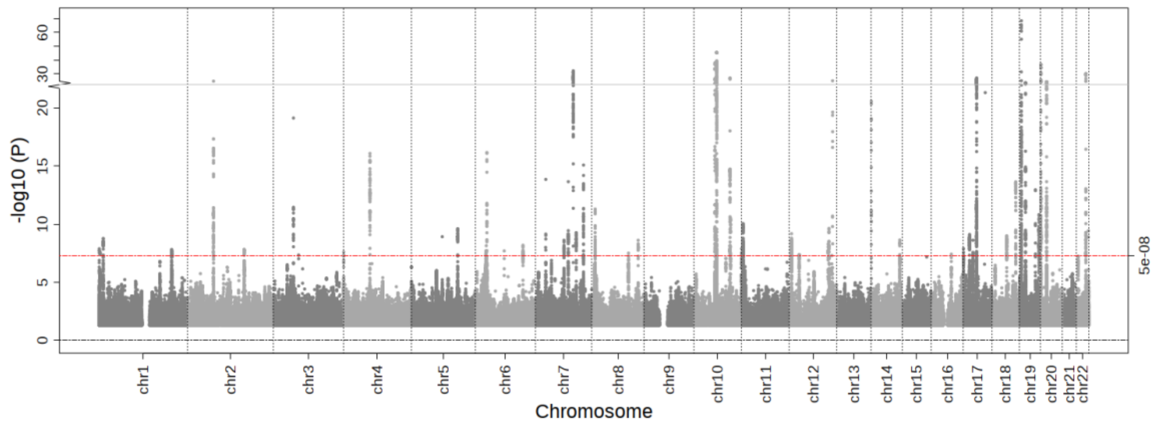

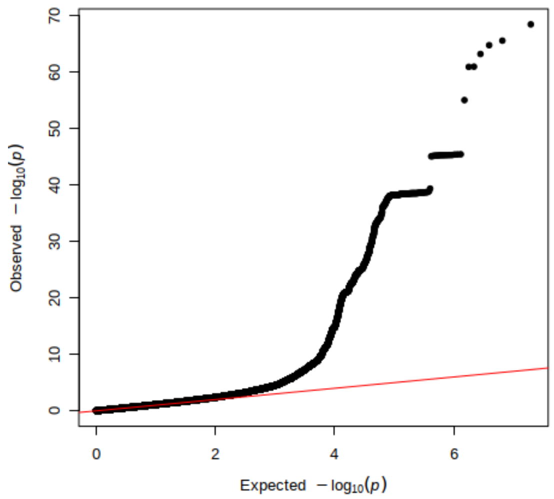

Figure 2 Manhattan plot and quantile-quantile (Q-Q) plots of genome-wide association studies for mtDNA copy number in UK Biobank. The Manhattan plot shows the -log 10 (observed P values) for association of SNPs (y-axis) plotted against their chromosomal positions on each chromosome (x-axis), and the horizontal red line depict the genome-wide significant (5e-8) threshold. For the QQ plot, the x-axis indicates the expected -log 10 -transformed P values, and the y-axis shows the observed -log 10 -transformed P values. Black points represent the genome-wide significant association.

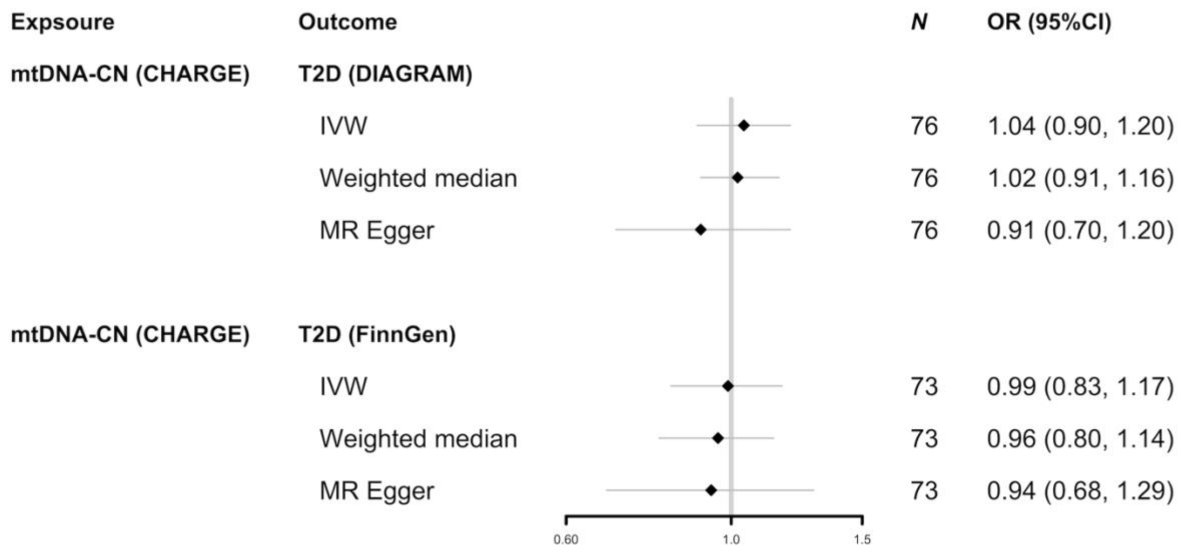

Figure 3 Forest plot for mtDNA-CN (CHARGE) and type 2 diabetes. N: number of SNPs analyzed in the MR analyses; OR: odds ratio; 95% CI: 95% confidence interval; IVW: inverse variance weighted.
